# A new gender-specific model for skin autofluorescence risk stratification

**DOI:** 10.1038/srep10198

**Published:** 2015-05-14

**Authors:** Muhammad S. Ahmad, Zoheir A. Damanhouri, Torben Kimhofer, Hala H. Mosli, Elaine Holmes

**Affiliations:** 1 Drug Metabolism Unit, King Fahad Medical Research Center, King Abdulaziz University, Jeddah, 21589, Saudi Arabia; 2 Department of Pharmacology, Faculty of Medicine, King Abdulaziz University, Jeddah, 21589, Saudi Arabia; 3 Section of Biomolecular Medicine, Division of Computational and Systems Medicine, Department of Surgery and Cancer, Imperial College London, SW7 2AZ, United Kingdom; 4 Department of Medicine, Faculty of Medicine, King Abdulaziz University, Jeddah, 21589, Saudi Arabia

## Abstract

Advanced glycation endproducts (AGEs) are believed to play a significant role in the pathophysiology of a variety of diseases including diabetes and cardiovascular diseases. Non-invasive skin autofluorescence (SAF) measurement serves as a proxy for tissue accumulation of AGEs. We assessed reference SAF and skin reflectance (SR) values in a Saudi population (n = 1,999) and evaluated the existing risk stratification scale. The mean SAF of the study cohort was 2.06 (SD = 0.57) arbitrary units (AU), which is considerably higher than the values reported for other populations. We show a previously unreported and significant difference in SAF values between men and women, with median (range) values of 1.77 AU (0.79–4.84 AU) and 2.20 AU (0.75–4.59 AU) respectively (*p*-value « 0.01). Age, presence of diabetes and BMI were the most influential variables in determining SAF values in men, whilst in female participants, SR was also highly correlated with SAF. Diabetes, hypertension and obesity all showed strong association with SAF, particularly when gender differences were taken into account. We propose an adjusted, gender-specific disease risk stratification scheme for Middle Eastern populations. SAF is a potentially valuable clinical screening tool for cardiovascular risk assessment but risk scores should take gender and ethnicity into consideration for accurate diagnosis.

Accumulation of advanced glycation endproducts (AGEs) is linked to aging and pathophysiology of a variety of diseases including diabetes, cancer, cardiovascular diseases and neurodegenerative disorders^1^,^2^,^3^,^4^,^5^,^6^. Advanced glycation endproducts are formed via the Maillard’s reaction between carbonyl groups of reducing sugars and free amino groups from proteins or other macromolecules. The accumulation of AGEs in the body leads to structural and functional modifications of tissue proteins, adversely affecting the function of those proteins^7^. Long lived proteins such as skin collagen, lens crystallins and cartilage proteins are more susceptible to AGEs accumulation over time due to their longer half-life^8^.

Some AGEs form protein-protein cross-links and certain species naturally exhibit fluorescence properties. Measurement of skin autofluorescence (SAF), is currently being tested as a non-invasive tool to estimate the degree of AGEs accumulation in the human body/skin^9^. SAF has been shown to provide a measure of cumulative metabolic stress and correlate with collagen-linked fluorescence and both, fluorescent (pentosidine) and non-fluorescent (N^ε^-(carboxymethyl)-lysine) AGEs. Several validation studies simultaneously measured SAF and specific AGEs from skin biopsy samples at the same measuring site, and reliably demonstrated that SAF is a good proxy for tissue accumulation of AGEs^9^,^10^,^11^.

SAF is calculated as the average light emission intensity in the 420–600 nm wavelength range after ultraviolet-A excitation with a peak maximum intensity of 370 nm. A confounding factor of SAF measurement is the degree of skin pigmentation, i.e., darker skin attenuates the detectable signal as it absorbs more light than lighter skin. Skin reflectance (SR) is a metric of the amount of light reflected by the skin, measured in the 320–420 nm wavelength range and is dependent upon skin pigmentation. To address the issue of the effect of skin pigmentation on SAF values, Koetsier *et al.* developed an adjusted algorithm that incorporates information about the degree of UV light absorption in the spectral regions of melanin^12^.

Increased skin concentrations of AGEs are associated with type 2 diabetes and have been shown to add diagnostic value to the conventional risk scheme for cardiovascular risk assessment in type 2 diabetes^13^. Furthermore, it has been reported that SAF based decision trees perform better in detecting diabetes or impaired glucose tolerance in intermediate-risk persons compared to the conventional method of assessing fasting plasma glucose and HbA1c^14^. Although SAF shows promising translational impact, it is still at the experimental and investigational stage. Further large, cross-sectional, case-controlled and case-series studies are needed to gather more SAF data to establish reference ranges in different ethnic populations and skin types before this technique can be widely used in primary care setting for the diagnosis and management of different diseases, especially diabetes. Several recent studies have tried to address this issue by providing SAF reference values for Dutch, Chinese, Slovak and Qatari populations^15^,^16^,^17^,^18^. However, all of these studies have highlighted the importance of gathering more SAF measurements in larger cohorts.

Obesity-related diseases are prevalent in Middle Eastern countries and Saudi Arabia is among the top ten countries with respect to prevalence of diabetes^19^. Thus, large-scale studies to establish reference SAF ranges in Arab populations may help in earlier diagnosis of diabetes in this region. To date, a recent study carried out in Qatar has been the only study to report SAF values in an Arab population, and in contrast to studies in other populations, suggested a gender discordance in SAF values^18^. We aimed to determine reference SAF values for the general Saudi population and compare it to other cohorts reported in the literature. A secondary objective was to ascertain whether the general Saudi population has higher SAF or steeper age-dependent rise compared to other cohorts, given the higher prevalence of diabetes. We examined whether gender, ethnicity, obesity, smoking, physical activity, hypertension and diabetes were correlated with SAF and explored the performance of the risk stratification scale with respect to high/low SR values.

## Results

### Baseline characteristics of skin reflectance and skin autofluorescence values in a Saudi population

Skin reflectance and SAF values were established for a total of 1,999 participants (median age 38 yrs (18–98 yrs)), of which 1,122 were men (56%, median age 39 yrs (18–98 yrs)) and 877 were women (44%, median age 38 yrs (18–80 yrs)). A summary of numerical and categorical data is presented in tabular form for this cohort (Tables 1 and 2, respectively). There was no significant difference in age between the two genders (Tables 1, *p*-value 0.08). Comparison of the whole cohort (n = 1,999) versus the healthy participants (n = 1,038) shows that in the healthy cohort, the ratio of men:women in the overweight and obese categories is approximately 1:1 (Supplementary Fig. S1), whereas there is a higher ratio of men:women in these categories in the full population (approximately 3:2). Saudi nationals constituted 57% of the whole cohort with a similar distribution of males (56%) and females (44%) as in the healthy cohort.

Mean SR was 9.5 (±3.0) percent and the majority of individuals had SR values below 11%. A weak but significant difference between SR values of males and females was observed, with median values of 8% and 9% respectively (Tables 1, *p*-value « 0.01). Mean SAF was 2.06 (±0.57) arbitrary units (AU) for the entire cohort. There was a significant difference in the SAF values of men and women, with median values of 1.77 AU (0.79–4.84 AU) and 2.20 AU (0.75–4.59 AU) respectively (Tables 1, *p*-value « 0.01).

As expected, skin autofluorescence directly correlated with age (Table 3, Supplementary Fig. S2) and despite the fact that women had significantly higher SAF values than men across the whole age range, with a gender effect size of 0.92 AU (95% CI: 0.83–1.02, *p*-value 2.2 × 10^−16^), the relationship between age and SAF was similar, as indicated by similar slopes in both linear regression models (Supplementary Fig. S2). The statistical models fitted for male and female participants of the healthy cohort (no hypertension, no diabetes) are:


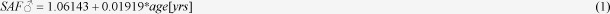



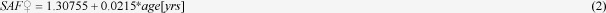


Further, we tested the relationship of SAF with a wide range of skin pigmentations to explore the validity of the implemented SR adjustment method in Middle Eastern populations. For that purpose, we categorised the study participants into two groups: high SR (>10%) and low SR (< =10%) and tested for group differences between SAF values for individuals with low and high SR (Supplementary Fig. S2). After correcting for the confounding factors age, body mass index (BMI) and diabetes, we found significant differences in SAF for women (*p*-value 9.5 × 10^−6^), but not for men (*p*-value 0.12). The mean standardized difference (MSD) was used to quantify the magnitude of the effect of SR on SAF. For men, the MSD was close to zero (no effect, both SR groups), whilst for women with high SR the MSD was 0.32 AU SAF (95% CI: −0.46 – −0.18). This gender discrepancy in SAF values has not been reported for other populations, with the exception of a single study in a Qatari population^18^. It further indicates that the correction factor used by the AGE reader is not appropriate for the women in this cohort as it overestimates their risk for AGE-related morbidities.

In order to take the effect of differential SR values in the female participants into account, we stratified the female sub-cohort into high and low SR groups and developed the following, SR-adjusted linear regression models:


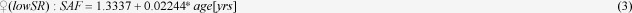



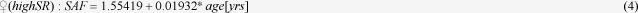


**Figure 1:**
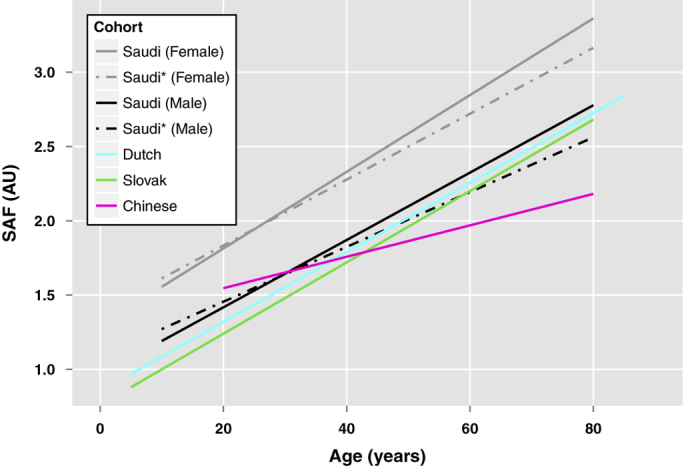
Comparison of SAF values between different cohorts. Saudi men (black) had similar SAF values to the Dutch’s (cyan) and Slovak’s (green). Increased SAF values have been observed for the Saudi women (grey). This relation was also true if the full cohort was reduced to a subset of healthy individuals, defined by the absence of diseases (dashed lines).

To highlight further population differences we established regression models (SAF vs age) for a healthy sub-population (defined by the absence of disease, as assessed by a questionnaire) and compared these with data reported for other cohorts (Fig. 1).

Over the entire age range (~18–75 years) the SAF values for healthy men were similar to those reported for Dutch^15^. and Slovak^17^. populations. Comparable SAF values have also been reported for Chinese individuals up to the 4^th^ life decade, although, in later ages they showed a less steep increase in SAF^16^. In contrast, the female participants of the Saudi cohort demonstrated considerably higher SAF values over the entire age range than all other cohorts (on average 0.5 AU higher than the Dutch). This observation is consistent with a Qatari study, where women had significantly higher SAF values than men across the whole age range, with a gender effect size of 0.42 AU (95% CI: 0.30 – 0.54, *p*-value < 0.001)^18^. The mean SAF values reported for the Qatari cohort (2.27 ± 0.63 AU) were higher than those found in the Saudi cohort (2.06 ± 0.57 AU). This difference is probably due to different age distributions in the two cohorts (median age 48 yrs and 38 yrs for the Qatari and Saudi cohort, respectively) and the inclusion of a relatively high number of individuals suffering from type 2 diabetes in the Qatari cohort (51% as opposed to 14% in the Saudi cohort).

**Figure 2:**
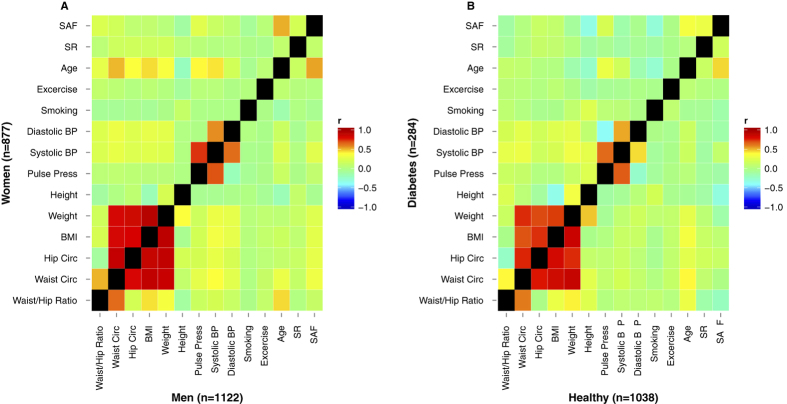
Correlation heatmaps stratified for gender and diabetes. (**A**) The upper triangular matrix shows correlations among variables for women, the lower triangular matrix for men. (**B**) The upper triangular matrix shows correlations among variables for diabetic subjects, the lower triangular matrix for non-diabetic subjects.

In addition to the observed association between SAF and age, the following variables showed strong correlations (as defined by Pearson’s correlation coefficient, *r*): weight, BMI, hip circumference and waist circumference (each *r* > 0.81), as well as systolic and diastolic blood pressure (*r* > 0.57) for both men and women. As expected the correlation of waist:hip ratio with waist circumference and BMI, (Fig. 2A) was stronger in men (*r* = 0.65 and *r* = 0.44, *r*espectively) than in women (*r* = 0.52 and *r* = 0.22, *r*espectively), whereas age was more strongly correlated with hip circumference and BMI in women than in men.

Smokers had median SAF values of 1.81 AU (1.04–4.0 AU) compared to non-smokers with median SAF values of 2.0 AU (0.75–4.84 AU). After correcting for the confounding factors age, BMI, diabetes and SR, however, no significant effect size remained for smoking in both genders (MSD close to zero). The same was true for the effect of physical activity.

### The influence of body mass index and waist to hip circumference on SAF

**Figure 3:**
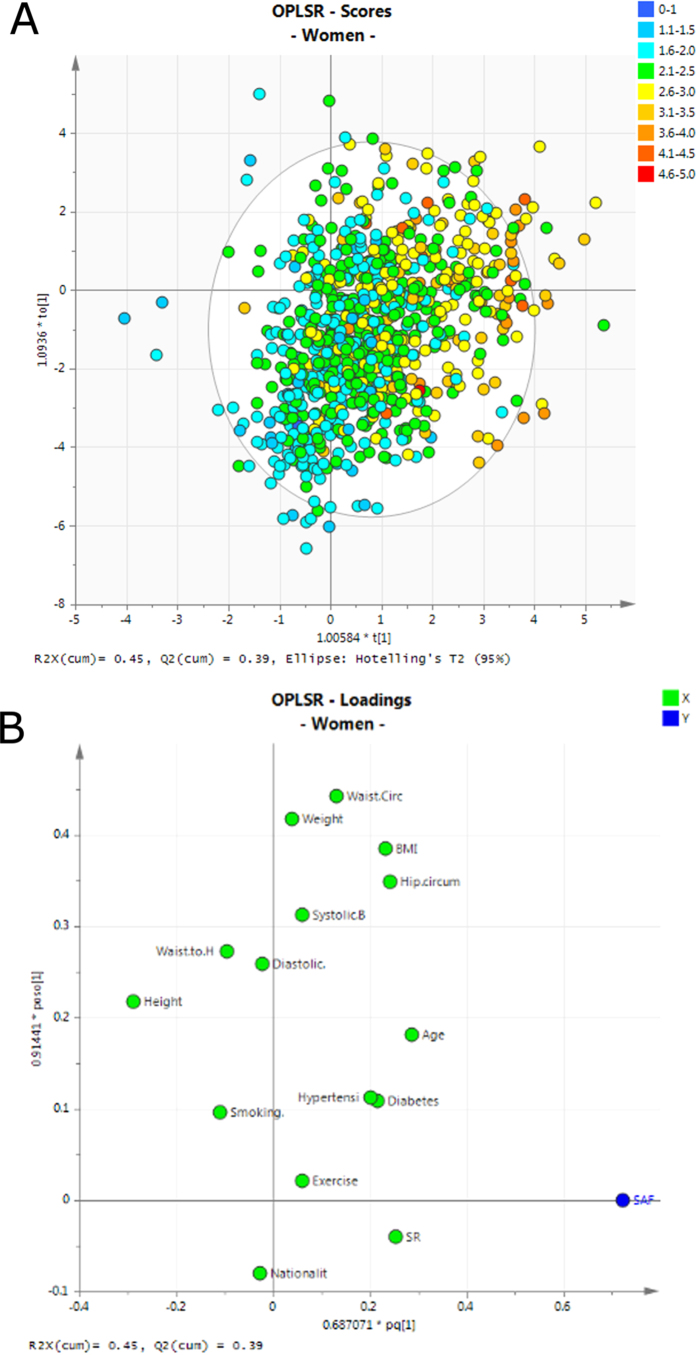
Orthogonal partial least squares regression (OPLSR) model for women. (**A**) Scores plot (**B**) Loading plot. Variables with high influence include age, diabetes, hypertension and SR. Hypertension and SR seems to be less influential for men (Fig. 4).

**Figure 4:**
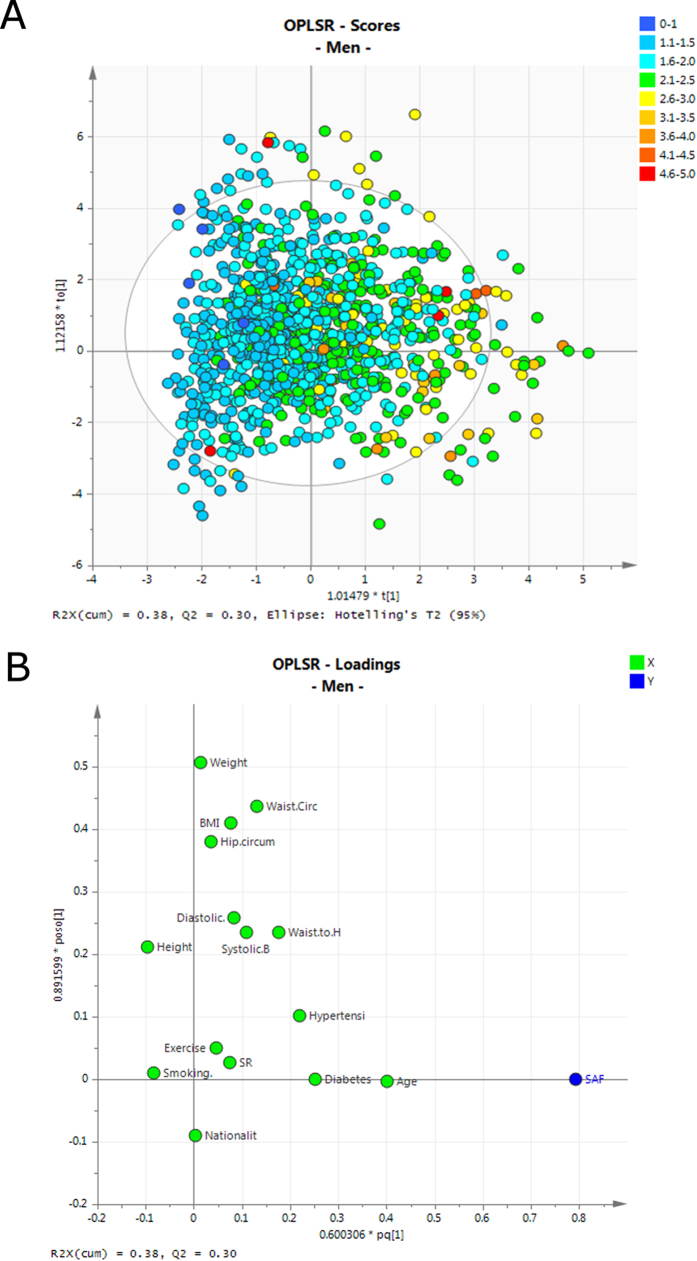
Orthogonal partial least squares regression (OPLSR) model for men. (**A**) Scores plot (**B**) Loading plot. Variables with high influence include age and diabetes. Hypertension and SR seems to be less important in men than in women (Fig. 3).

A high level of obesity (as defined by the WHO for BMI > 30) was found in this cohort where the average BMI was 30.24 (± 6.28) and the median BMI was 30. No significant difference in BMI was found between men and women (Table 1; *p*-value, 0.74). Only 20% of the cohort had a normal or low weight (BMI < 25) and the remaining individuals were either overweight (33%, BMI > = 25 and < 30) or obese (47%, BMI > = 30). More men were overweight or obese than women (47% vs 33%, respectively) as shown in Supplementary Fig. S1. This is in contrast to WHO country profile for Saudi Arabia, which reported that 33% of the population is obese with higher obesity rates in women (39.1%) compared to men (28.6%). The median SAF values were 1.78 AU (1.13–2.37 AU) in under-weight individuals, 1.89 AU (0.75–4.84 AU) in normal weight individuals, 1.91 AU (0.79–4.59 AU) in over-weight individuals and 2.04 AU (0.86–4.72 AU) in obese individuals. Although the differences in median SAF values seem to be striking, many of them diminish after correcting for the known covariates age, gender, diabetes and SR. After correction, there remained a significant SAF difference in overweight men, when compared to men whose weight was in the normal range, with an average increase of 0.32 AU (95% CI: 0.13–0.50, *p*-value 0.003). This difference was only observable for male and not for female participants. However, multivariate modelling with orthogonal partial least squares regression (OPLSR), which takes the synergistic variable effects into account clearly shows a dependency of SAF on waist to hip ratio for both women and men (Figs. 3 and 4 respectively), with BMI having a high model importance in women, validated with permutation resampling (n = 999 iterations; Supplementary Fig. S3).

### Gender dependent associations between SAF and diabetes

**Figure 5:**
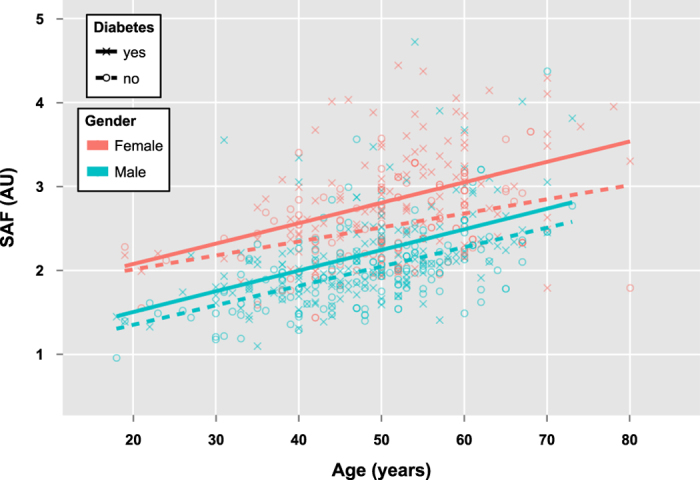
SAF stratified for diabetes and gender. In Saudi men (blue) and women (red), diabetic subjects (solid lines) had increased SAF values when compared to non-diabetic subjects (dashed lines). In later age stages, this difference seems to be more pronounces in women than in men.

We directly compared the subset of people with diabetes (n = 284) with an equal-sized subset of age, gender and BMI matched controls (n = 284). Female and male diabetic subjects had significantly higher SAF values across the entire age range than their non-diabetic counterparts (Fig. 5). The association between SAF value and age was diminished in diabetic subjects (*r* = 0.36) compared to healthy participants (*r* = 0.46). Other correlations between variables that were discordant for diabetic subjects and healthy participants (Fig. 2B) were a positive association between waist circumference and waist hip ratio (diminished in diabetic subjects), an inverse correlation between diastolic blood pressure and pulse pressure (stronger in diabetic subjects) and an inverse association between height and BMI (stronger in diabetic subjects). The effect size for non-diabetes/diabetes for men 0.41 AU (95% CI: 0.24–0.58. *p*-value 5.0 × 10^−5^) was slightly lower than the one for women, 0.53 AU (95% CI: 0.34–0.73, *p*-value 1.5 × 10^−5^). OPLSR models confirmed the positive association between diabetes and SAF value in both women and men (Figs. 3 and 4 respectively).

### Association of hypertension with SAF

Based on the categorical data indicating whether participants were hypertensive (assessed by a participant questionnaire), a small but non-significant effect of hypertension on SAF (SMD = 0.126 AU, 95% CI: 0–0.25, *p*-value 0.072) was found. This effect was diminished if only patients that were hypertensive at the time (with systolic BP > 160 and diastolic BP > 100) were included, thereby taking into account the effect of medication. However, the OPLSR model shows that hypertension was a significant predictor of SAF values for both women and men and co-mapped with self-reported diabetes (Figs. 3 and 4 respectively).

### Adjusted scheme for CVD risk stratification

**Figure 6:**
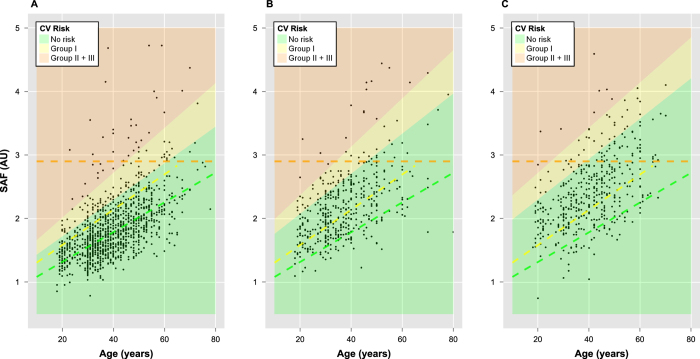
Proposed CVD risk stratification for Saudi cohort. (**A**) Men (**B**) Women with low SR values and (**C**) Women with high SR values. The background colour indicates the predicted risk group. The boundaries for the original risk stratification groups by Koetsier *et al.* are indicated by the dashed lines where the red dashed line represents the cut off for the highest risk group for CVD. Based on the original boundaries, it can be seen that the majority of women participants with high SR values (**C**) sub-cohort are allocated to the risk groups II and III.

**Figure 7:**
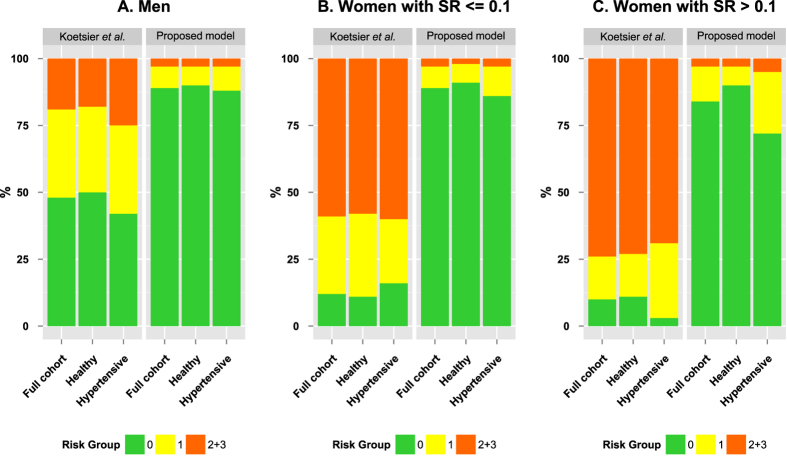
Comparison of risk score distributions for Saudi cohort. The original model developed by Koetsier *et al.* and the new proposed model are compared for (**A**) Men, (**B**) Women with low SR values and (**C**) Women with high SR values.

Using SAF values to stratify an individual’s risk of developing cardiovascular disease (CVD) is a major application of the AGE reader. Risk stratification is based on the initial study and validation of the method in a Dutch cohort by Koetsier *et al.*^15^, whereby individuals are assigned a CVD risk group 1–3. The Saudi cohort shows significant gender differences in SAF compared to other cohorts (Fig. 1), and the SR value influences the SAF value in women despite the adjusted algorithm (Supplementary Fig. S2). The majority of women, especially with higher SR in Saudi cohort are classified to be at higher CVD risk according to Koetsier *et al.* risk stratification model (Figs. 6 and 7). However, this is not supported by the data for other known risk factors of CVD, i.e., hypertension, overweight/obesity, diabetes, cholesterol, tobacco use and lack of physical activity in our study, where there is no marked gender effect. Moreover, the CVD risk score proposed by Koetsier’s model is not consistent with the mortality incidence reported in the literature for Saudi population (23.5% for Saudi women and 27% for Saudi men)^20^. Therefore, we propose the following, adjusted risk stratification schemes for Middle Eastern populations, stratified for gender and SR (Fig. 6):

**Risk group 0: (No CVD risk < mean SAF + 1SD):**


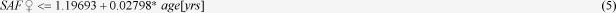



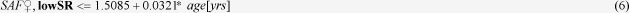



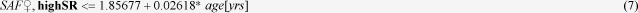


**Risk group 1: (limited increase of CVD risk > mean SAF + 1SD AND < mean SAF + 2SD):**


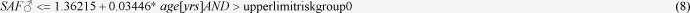



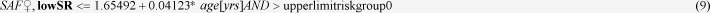



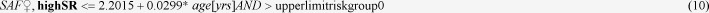


**Risk group 2 or 3: (increased or definite CVD risk > mean SAF + 2SD):**


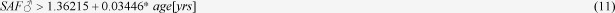



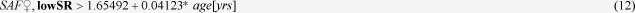



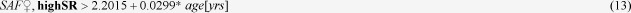


A comparison of risk score distributions generated from the conventional and proposed scheme for Saudi cohort is illustrated in Fig. 7. By using the conventional model for women with high SR (the most extreme group), 235 participants (74%) were assigned to the medium and high risk categories (2 and 3), whereas using the modified risk stratification developed here only 10 participants (3%) were assigned to the medium to high risk categories (Fig. 7C). Similarly, when conventional algorithm was applied to healthy Saudi cohort (women with high SR), 118 participants (73%) were assigned to the medium to high risk categories (2 and 3), whereas using the modified risk stratification developed here only 4 participants (3%) were assigned to the medium to high risk categories. Similarly, the conventional model over-estimated the CVD risk for women with low SR and men in the full current cohort, healthy sub-cohort and hypertensive sub-cohort that is corrected in the proposed model (Fig. 7).

## Discussion

We provide reference SAF data for the first time in a Saudi population with potential application in a wide-range of translational and clinical studies. Skin autofluorescence assessment is not only important for cardiovascular risk assessment^13^,^21^,^22^ and diabetes screening^14^, but has also been shown to have diagnostic value in renal failure 23,24,25, retinopathy^26^,^27^, neuropathy^28^,^29^, foot ulceration^30^,^31^,^32^ and schizophrenia^33^. We show a gender dependent association of SAF with age, SR value, diabetes, hypertension and obesity, discussed below. Half of our cohort was physically active (reported exercising for more than 30 minutes per day) and approximately 20% were smokers. We found no association of SAF with smoking or exercise. These results are contrary to previous reports, that associate increased SAF values with smoking^34^, particularly in cohorts with central adiposity^35^ and a decrease of SAF in physically active individuals^17^, although other studies have found no such association between smoking and SAF values^36^.

### Gender bias in SAF values

The mean SAF value of the study cohort was 2.06 AU (SD, 0.57), which is considerably higher than the values reported for other populations. We found a significant gender difference in SAF values, where women had higher SAF values compared to men. This is in contrast to most other studies that indicate no gender difference in SAF values in Dutch^15^, Chinese^16^ and Slovak populations^17^. One previous study reported 0.2 AU higher SAF values in female smokers, compared to male smokers (*p*-value 0.02) but no gender difference in SAF values was observed in non-smoking subjects^15^. Similar gender differences in SAF were reported in a recent Qatari study, with a cohort made up of mainly Arabs (57%), South Asians (28%) and Filipinos (10%)^18^. According to WHO country profile, cardiovascular mortality is higher in Saudi males accounting for ~14% of all deaths due to non-communicable diseases between the ages of 30-70 years compared to Saudi females (~7%)^37^, therefore the higher SAF values observed in females is unlikely to be a true representation of higher cardiovascular risk. The majority of our cohort were Arab individuals similar to Qatari cohort, thus the gender difference in SAF values among Arabs may be attributable to genetic contributions.

Some previous studies suggested that genetics play a role in skin and lens fluorescence^38^,^39^. Indeed, a recent genome-wide association study has reported an association between *N*-acetyltransferase 2 (NAT2) acetylation status and SAF in people with and without diabetes of European decent^40^. However, the male participants in our cohort had similar SAF values to age matched Dutch, Slovak and Chinese individuals, and no sex-chromosome linked associations have been reported so far.

Women in the Middle Eastern region keep their arms covered due to cultural reasons and usually have a lighter skin colour than males. Indeed, females in our cohort had significantly higher SR than males (median SR: 9% and 8%, respectively), consistent with lighter skin colour. The Middle East has the highest level of vitamin D deficiency in the world^41^, and the prevalence is higher in women than in men^42^, largely attributable to lifestyle and behavioural differences between genders. Consequently this may partially explain the higher SAF values found in the Saudi women, since vitamin D deficiency is associated with diabetes and obesity. Further studies are needed to determine the association between vitamin D deficiency and SAF in Saudi women. An alternative explanation for gender differences in SAF may be the residual effect of application of skin creams, lotions and sunscreen^43^, predominantly by women. Skin products can have fluorescent properties that may serve to produce a cumulative effect to increase the SAF signal in women. However, the evaluation phase of our study indicates that the effect of skin lotion on SAF values is vastly reduced by a single wash using soap and water (Supplementary Table S1) and this, together with the fact that participants were excluded from the main study if they had applied lotions or skin products within 48 hours prior to the study, would suggest that this explanation does not in itself account for the observed differences. Further, appropriately controlled studies are needed to investigate this phenomenon.

Within the female subset, based on both univariate and multivariate analyses, we found that women with darker skin colour (SR ≤ 10%) had lower SAF measurements than those with lighter skin colour (SR > 10%). This is an unexpected finding as we used the SR independent SAF measurement software for SAF assessment. Since there was no SR effect in men, which had considerably smaller SAF values, we conclude that the implemented correction method for SR may not be appropriate for individuals with SAF values higher than the reference values proposed by Koetsier *et al.*^12^. This also implies that the SAF modelled risk score is underestimated for subjects with SR ≤ 10% at high risk of developing cardiovascular disease. Similarly, risk scores were overestimated for individuals with higher SAF values (related to higher SR). Therefore, we suggest that the cardiovascular risk assessment provided by the AGE reader may need to be revised for different ethnic populations and have proposed a modified risk stratification algorithm.

### SAF association with cardiovascular disease co-morbidities

In the current study, only 52% of the cohort, comprising 1,038 individuals was defined as healthy, i.e., no self-reported disease, with systolic BP of 100-139 and diastolic BP of 50-89 millimetres of mercury. This compares poorly with other random catch population studies reporting SAF values (Dutch, Slovak and Chinese) where the incidence of obesity in those countries is 18.8%, 25.4%, and 5.7%, respectively^37^. Nevertheless, the SAF values for the subset of Saudi men were similar to these other populations. The Qatari population was the most closely matched to the Saudi population in terms of prevalence of diabetes, obesity etc. and also reported a similar gender bias that did not correlate with mortality from CVD^37^.

Higher SAF values were observed in overweight and obese individuals based on the OPLSR models, which showed that waist-to-hip ratio in men and both BMI and hip circumference in women were strong coefficients in the equation (Fig. 3). These observations are in agreement with other literature that reports higher mean SAF values in centrally obese individuals compared to non-obese individuals (*p*-value 0.001)^35^. We find an SAF increase of 0.15 AU in obese people compared to normal weight people in our study similar to the study by den Engelsen *et al.*, who reported a SAF increase of 0.13 AU in a sub-group of individuals with central obesity compared to the sub-group of people without central obesity, after adjusting for age and smoking^35^.

We report for the first time, a direct association between SAF value and the presence of hypertension. The coefficient plots for the OPLSR model show co-mapping of hypertension and diabetes as factors that contribute to higher SAF values (Figs. 3 and 4). Obesity is an acknowledged and independent risk factor for CVD, hypertension and diabetes^44^. Vitamin D deficiency has been linked with cardiometabolic dysfunction (e.g., hypertension, insulin resistance, type 2 diabetes mellitus, obesity, and dyslipidemia)^45^. Although we proposed vitamin D deficiency as a possible reason for gender bias in SAF reading, the CVD mortality statistics, which are worse for men, do not support vitamin D deficiency as a reason for increased SAF levels.

The conventional classification of CVD risk, based on SAF data from Koetsier *et al.* in a Caucasian population, is clearly not applicable to the Saudi population since it places 334 (59%) women with low SR and 235 (74%) women with high SR of our study cohort in high risk groups (2 and 3). Even for the healthy sub-cohort, it massively overestimates the CVD risk by assigning 181 (58%) women with low SR and 118 (73%) women with high SR into high risk categories. This is not supported by the data from epidemiology studies and identifies the need to adjust the risk stratification scheme based on reference SAF values in this ethnic population. We revised the risk stratification for both men and women (with high and low SR) to match the documented mortality associated with cardiovascular disease in the Saudi population. This was achieved by setting the upper SAF limits for risk group 0 and 1 to the mean SAF plus one and two standard deviations per age group, respectively. This resulted in down-weighting of individuals, especially women from the high risk to lower risk category in the full cohort and in both healthy and hypertensive sub-cohorts (Fig. 7). Validation of this modified risk stratification scheme is now warranted in other Middle Eastern populations.

Limitations of our study include that we did not recruit subjects from populations outside Jeddah, however, Jeddah is a multi-ethnic city with people from different Middle Eastern countries and around the world. We did not perform tests for glucose or HbA1c. Many subjects may have pre-diabetes or undiagnosed diabetes which may have a pronounced effect on the average SAF values reported for this population. Since this study was conducted in public places, it was not possible to conduct blood tests for practical reasons. Moreover, at our study sites, it would not have been possible to find people in fasting state to perform reliable glucose testing. We are aware that we did not gather data about duration of diabetes in diabetic subjects. We also did not screen for renal function and smoking status, other likely contributors to SAF. Since the study population was relatively large (n = 1999), for reasons of practicality we were reliant on self-reported data. We might have excluded subjects with self-reported diseases, however, we also present data for a healthy sub-cohort.

In summary, we provide reference SAF values for the first time in a cross-section of Saudi population. Besides an increase of SAF with age, which has been described previously, we observed higher SAF values in women than in men. Therefore, we advocate that gender differences should be taken into consideration in developing and implementing SAF-based risk engines for disease screening and/or monitoring. Reference SAF values must be established in different ethnic populations before SAF can be used in clinical settings. Further larger studies are needed in different ethnic groups and different geographic locations to understand the contribution of genetic variations, environmental factors and different life styles to the variance in SAF.

## Methods

### Study design

The cross-sectional study was approved by the research ethics committee of King Abdulaziz University. All procedures were carried out in accordance with the approved guidelines. Bioethical regulations as described in the “Regulations of Research Bioethics on the living Creature” by the National Bioethics Committee (King Abdulaziz City for Science and Technology) were strictly observed by all persons associated with the project while dealing with the study subjects. A questionnaire was used to gather information about demographical variables (age, gender), ethnicity (country of birth, nationality), lifestyle factors (smoking habits, physical activity) and medical history (presence of age related diseases in the subject and his/her first degree relatives and medicine taken). All participants had physical examination to measure height, weight, hip circumference, waist circumference, blood pressure, skin reflectance (SR) and skin autofluorescence (SAF). All adults were invited to take part in the study who could communicate in Arabic or English. The subjects who provided written informed consent were included in the study. Subjects below the age of 18 or with Fitzpatric class 5-6 skin type were excluded from the study.

### Study population

This study was performed at various locations including King Abdulaziz University, King Abdulaziz University Hospital, Al-Salaam Mall and Mall of Arabia between November 2013 and April 2014 in the city of Jeddah, the largest urban center in the western Saudi Arabia. A total of 2,385 subjects provided the informed consent and completed the questionnaire. However, 239 subjects couldn’t wait and left the stall before their SR and SAF was measured (The same AGE reader was used for all subjects). Among the remaining 2,146 subjects who completed the procedure, valid SAF measurement was not possible in 147 subjects due to skin darkness. Therefore, data are presented for 1,999 individuals. There was no statistical difference in the demographics of those participants who left or were excluded from the study before completing the full set of measurements and those who completed the study regardless.

### Skin autofluorescence measurement

The SAF measurements were performed non-invasively using a fully automated AGE reader with a built-in spectrometer (DiagnOptics Technologies B.V., Groningen, the Netherlands) which has been described in more details previously^16^. The output of the AGE reader was a SR corrected SAF value as described in details previously^12^. Three measurements were made for each participant by slightly changing the position each time at healthy intact skin on volar side of dominant forearm approximately 5-10 cm below elbow fold. Average SAF value was calculated by the AGE reader software version 2.3, which performs a correction to the SAF value if the UV reflectance from skin is between 6% and 10%. However, valid SAF measurement for skin with UV reflectance below 6% is not possible with the current AGE reader SU software used in our study. AGE reader uses UV-A light with a peak excitation wavelength at 370 nm to illuminate ~4 cm^2^ skin area. Skin autofluorescence is calculated as the ratio between emission light intensity in the 420–600 nm wavelength range and the excitation light intensity in the 300–420 nm wavelength range, mulitiplied by 100 and is expressed in arbitrary units. Before taking the measurements, participants were asked about the use of tanning agents, creams, lotions or perfumes on the forearm within the previous 48 hours and if they had used such agents, SAF measurement was not performed for them. They were asked to wash the forearm thoroughly with soap, refrain from using such agents before measurement and come back after two days. We observed the effect of skin lotion application and subsequent washing with soap and water on SAF and SR values in a pilot study (Supplementary Table S1). We show that application of skin lotion increases SAF value by 18% and decreases SR value by 11%, consistent with the results reported previously^43^. Washing with soap and water reduces the effect of skin lotion on SAF and SR values by 62% and 65% respectively.

Reproducibility and robustness of AGE Reader measurements is already well-established^46^,^47^,^48^. For example, McIntyre *et al.* report a coefficient of variation of 8% for different operators and 7% for repeat measurements performed on a single subject by the same operator^48^. We conducted a pilot study to gather data about intra-personal and operator variance and found both, the SAF and the SR measurements, to be robust with low inter-operator (CV: 3.9% and 4.6%, respectively) and intra-personal (CV: 6.2% and 8.1%, respectively) variance (Supplementary Tables S2–S4).

### Statistical analysis

Numeric data are expressed as mean (±SD) if normally distributed and as median (min-max) if non-normally distributed. The Shapiro-Wilk test was used to test for normal distribution (alpha = 0.05). Testing for population differences was accomplished with 2-sided t-testing, or, if the data violated the assumptions of normal distribution and homoscedasticity, with 2-sided Wilcoxon rank-sum testing. Categorical data were expressed as counts and population differences were tested with the chi-square test.

Correlation analysis was performed with Pearson’s correlation and heatmaps were generated to visualize correlation coefficients for gender and diabetes individually. The upper triangular matrix of Fig. 2A depicts correlations for women and the lower triangular matrix correlations for men. Similarly in Fig. 2B, the upper triangular matrix depicts correlation for individuals with diabetes and the lower triangular matrix correlations for non-diabetic subjects.

The comparison of SAF reference values across cohorts was based on linear regression models, established with mean SAF values per age group that were extracted from the following publications: Dutch study^15^, Chinese study^16^ and Slovak study^17^.

The effect size of covariates are expressed as standardized mean differences (SMD, sometimes known as Cohen’s d), after adjusting the outcome variable SAF for confounding factors. For example, the effect size of the variable *gender* on SAF was calculated after removing the influence of confounding factors age, diabetes, BMI and SR by means of linear regression:


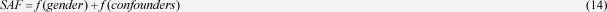



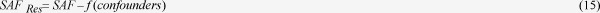


with 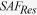 being the residuals of a linear regression model established with the confounding factors age, diabetes, BMI and SR. The size effect was calculated with the following formula:


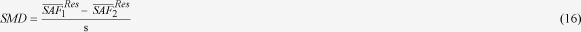


Whereas 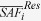 is the average value of the corrected SAF for group 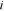 (in the example above: male and female) and s is the pooled standard deviation:


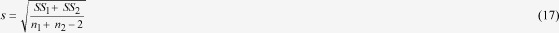


With 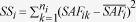 and 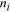 is the number of observations in group 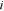. The 95% confidence intervals (CI) were estimated with a non-central t-distribution parameter as implemented in the R package *MBESS* (V 3.3.3), that is available via CRAN repository (http://cran.r-project.org/).

To assess whether SAF values can be predicted and to improve our understanding of the relationships among the predictor variables an orthogonal partial least squares regression (O-PLSR) model 49 was trained for each gender separately. PLSR is a multivariate data modelling technique that is particularly helpful in situations where there are multiple predictors and where these are (multi-) collinear or non-independent. The method combines features from principal component analysis and multiple regression such that, the input data (e.g. age, BP, BMI, weight etc.) and outcome measure (SAF) are described by latent variables. These latent variables (also termed principal components) represent composites of the original variables and are chosen in a way that maximises the covariance between them. OPLSR is an extension of the traditional PLSR approach which has been shown to yield more parsimonious models that are easier to interpret^49^. Similar to other modern predictive modelling techniques, (O)PLSR can easily overfit the data, which is why the number of principal components is usually determined with statistical re-sampling techniques. The presented OPLSR analysis was performed with the software SIMCA (V13), which produces model performance measures in form of Q^2^Y (estimated prediction accuracy of the model) and R^2^X (explained variance of the combined predictors) based on seven-fold cross validation. Both measures maximally take values of 1. However, for biological models the R^2^X and Q^2^Y usually have much lower values due to noisiness of the data and high inter-sample variation. The robustness of both models was tested by permutation analysis, where the sequence of the SAF values is shuffled in order to evaluate the likelihood of achieving similar high model R^2^X and Q^2^Y values by chance. We consider both of the trained OPLSR models (male and female) to be statistically robust (Supplementary Fig. S3), due to considerably smaller R^2^X and Q^2^Y values in the permutation models.

The optimal number of components were chosen according to a cross validation scheme. The OPLSR model for women showed similar and reasonably good predictive abilities (2 components, R^2^X = 0.45, Q^2^Y = 0.39) than the OPLSR model for men (2 components, R^2^X = 0.38, Q^2^Y = 0.30). The proposed scheme for adjustment of the CVD risk factor stratification was based on linear models established with the mean SAF values and standard deviations per age and gender group.

With exception of OPLSR analysis, all computation were performed with the statistical programming language R (V 2.15.2) on a 64 bit operating system running a Windows 7 distribution.

## Author Contributions

M.S.A., Z.A.D. and H.H.M. conceived and designed the study, obtained ethics approval and arranged resources/materials/analysis tools. M.S.A. collected data. M.S.A., T.K. and E.H. carried out data analysis and prepared the manuscript. T.K. preformed statistics and prepared all figures and tables. All authors reviewed and approved the final manuscript.

## Additional Information

**How to cite this article**: Ahmad, M. S. *et al.* A new gender-specific model for skin autofluorescence risk stratification. *Sci. Rep.* doi: **5**, 10198;10.1038/srep10198 (2015).

## Supplementary Material

Supplementary Information

## Figures and Tables

**Table 1:** Overview of numerical data

	**Men (n = 1,122)**	**Women (n = 877)**	***p*****-value**
**Age (yrs)**	39 (18 - 98)	38 (18 - 80)	0.08 (ns)
**Height (m)**	1.70 (1.48 - 1.92)	1.56 (1.22 - 1.86)	« 0.01
**Weight (kg)**	86.4 (42.6 - 160.0)	72.5 (35.2 - 135.7)	«0.01
**BMI (kg/m**^**2**^)	30 (16 - 55)	30 (15 - 70)	0.74 (ns)
**Waist circumference (cm)**	101 (58 - 166)	94 (56 - 140)	« 0.01
**Hip circumference (cm)**	107 (72 - 152)	109 (59 - 183)	0.02
**Waist to Hip Ratio**	0.95 (0.70 - 1.24)	0.87 (0.53 - 1.4)	« 0.01
**Diastolic BP (mm Hg)**	79 (51 - 182)	75 (25 - 157)	« 0.01
**Systolic BP (mm Hg)**	131 (99 - 211)	121 (85 - 225)	« 0.01
**Skin Reflectance (%)**	8 (6 - 33)	9 (6 - 33)	« 0.01
**SAF (AU)**	1.77 (0.79 - 4.84)	2.20 (0.75 - 4.59)	« 0.01

**Table 2:** Overview of categorical data.

	**Men** (n = 1122)	**Women** (n = 877)	***p*****-value**
**Smoking**	Yes = 328, No = 794	Yes = 64, No = 813	« 0.01
**Diabetes**	Yes = 163, No = 959	Yes = 121, No = 756	0.68 (ns)
**Exercise**	No = 530,	No = 464,	« 0.01
	1–2 times/month = 142,	1–2 times/month = 88,	
	1–2 times/week = 207,	1–2 times/week = 156,	
	3 or more times/week = 243	3 or more times/week = 169	
**Nationality**	Saudi Arabian = 640, Yemeni = 137,	Saudi Arabian = 503, Yemeni = 134,	« 0.01
	Egyptian = 111,	Egyptian = 50,	
	Jordanian = 49, Indian = 42, Pakistani = 38, Syrian = 36, Palestinian = 21,	Palestinian = 48,	
	Others^1^ = 48	Pakistani = 31, Syrian = 25, Jordanian = 19,	
		Philippine = 12, Indian = 11, Others^1^ = 44	
**Country of Birth**	Saudi Arabia = 712,	Saudi Arabia = 595,	« 0.01
	Egypt = 110, Yemen = 95, India = 41, Syria = 38, Pakistan = 36, Jordan = 32, Palestine = 13, Others^2^ = 45	Philippines = 12, India = 11, Morocco = 10, Others^2^ = 45	

^1^Afghan, Algerian, Bangladeshi, Canadian, Eritrean, Ethiopian, French, Indonesian, Lebanese, Moroccan, Romanian, Somali, Sudanese, Turkish, Turkistani, Uzbekistani, American, British, Burmese, Chadian, Dutch, Italian, Kenyan, Malaysian, Philippine, Russian, Spanish, Sri Lankan, Tunisian, Ugandan.

^2^Afghanistan, Algeria, Eritrea, Ethiopia, Indonesia, Jordan, Kuwait, Lebanon, Libya, Malaysia, Qatar, Romanian, Sudan, Turkey, United Arab Emirates, United Kingdom, United States of America, Bangladesh, Burma, Chad, Italy, Kenya, Morocco, Netherlands, Philippines, Somalia, Spain, Sri Lanka, Tunisia, Uganda.

**Table 3:** Average SAF values stratified by age and gender.

**Age [years]**														
	**<19**		**20-29**		**30-39**		**40-49**		**50-59**		**60-69**		**70-99**	
**Gender**	M	F	M	F	M	F	M	F	M	F	M	F	M	F
**N**	47	44	190	190	397	284	294	223	143	102	42	28	9	4
**Mean SAF (AU)**	1.42	1.84	1.59	1.99	1.75	2.19	1.98	2.51	2.22	2.76	2.44	2.87	2.75	3.19
**SD**	0.24	0.41	0.38	0.38	0.42	0.43	0.41	0.54	0.53	0.59	0.59	0.66	0.54	0.97
