# Corrigendum: A new gender-specific model for skin autofluorescence risk stratification

**DOI:** 10.1038/srep46832

**Published:** 2017-05-31

**Authors:** Muhammad S. Ahmad, Zoheir A. Damanhouri, Torben Kimhofer, Hala H. Mosli, Elaine Holmes

Scientific Reports 5: Article number: 10198; 10.1038/srep10198 published online: 05 14 2015; updated: 05 31 2017.

The Acknowledgements section in this Article is incomplete.

“We would like to express our gratitude to all the participants of the study, management of both King Fahad Medical Research Center (KFMRC) and Arabian centers, and all the students who volunteered to help us in recruiting the subjects. We thank all the support staff of department of Pharmacology, King Abdulaziz University Hospital, Al-Salaam mall and Mall of Arabia. We are particularly grateful to both Iuliana Denetiu and Mohammed Ali Majrashi for their help in collection, input and validation of data. We express our appreciation towards Hussain Ahmad for his help towards database development. We acknowledge the Stratified Medicine Graduate Training Programme in Systems Medicine and Spectroscopic Profiling (STRATiGRAD) for funding of Torben Kimhofer. The authors declare that there are no conflicts of interest.”

should read:

“This project was funded by the Deanship of Scientific Research (DSR) at King Abdulaziz University, Jeddah, under grant no. 2-141-35-HiCi. The authors, therefore, acknowledge with thanks DSR for technical and financial support. We would like to express our gratitude to all the participants of the study, management of both King Fahad Medical Research Center (KFMRC) and Arabian centers, and all the students who volunteered to help us in recruiting the subjects. We thank all the support staff of department of Pharmacology, King Abdulaziz University Hospital, Al-Salaam mall and Mall of Arabia. We are particularly grateful to both Iuliana Denetiu and Mohammed Ali Majrashi for their help in collection, input and validation of data. We express our appreciation towards Hussain Ahmad for his help towards database development. We acknowledge the Stratified Medicine Graduate Training Programme in Systems Medicine and Spectroscopic Profiling (STRATiGRAD) for funding of Torben Kimhofer. The authors declare that there are no conflicts of interest.”

