# Supplementary material for: A new gender-specific model for skin autofluorescence risk stratification
Source: Sci Rep. 2015 May 14;5:10198. doi: 10.1038/srep10198 (PMC4431468; doi:10.1038/srep10198)
Supplement: Supplementary Information [file srep10198-s1.pdf]

Supplementary material for:

A new gender-specific model for skin autofluorescence risk stratification.

Muhammad S. Ahmad[1*], Zoheir A. Damanhouri[1, 2†], Torben Kimhofer[3†], Hala H. Mosli[4], Elaine Holmes[1, 3]

[1]Drug Metabolism Unit, King Fahd Center for Medical Research, King Abdulaziz University, Jeddah, 21589, Saudi Arabia.

[2]Department of Pharmacology, Faculty of Medicine, King Abdulaziz University, Jeddah, 21589, Saudi Arabia.

[3]Section of Biomolecular Medicine, Division of Computational and Systems Medicine, Department of Surgery and Cancer, Imperial College London, SW7 2AZ, United Kingdom.

[4]Department of Medicine, Faculty of Medicine, King Abdulaziz University, Jeddah, 21589, Saudi Arabia.

†Both authors contributed equally to this study.

[*]To whom correspondence should be addressed. E-mail: maahmad2@kau.edu.sa

This File contains:

Figures S1-S3

Tables S1-S4

Supplementary Figures:

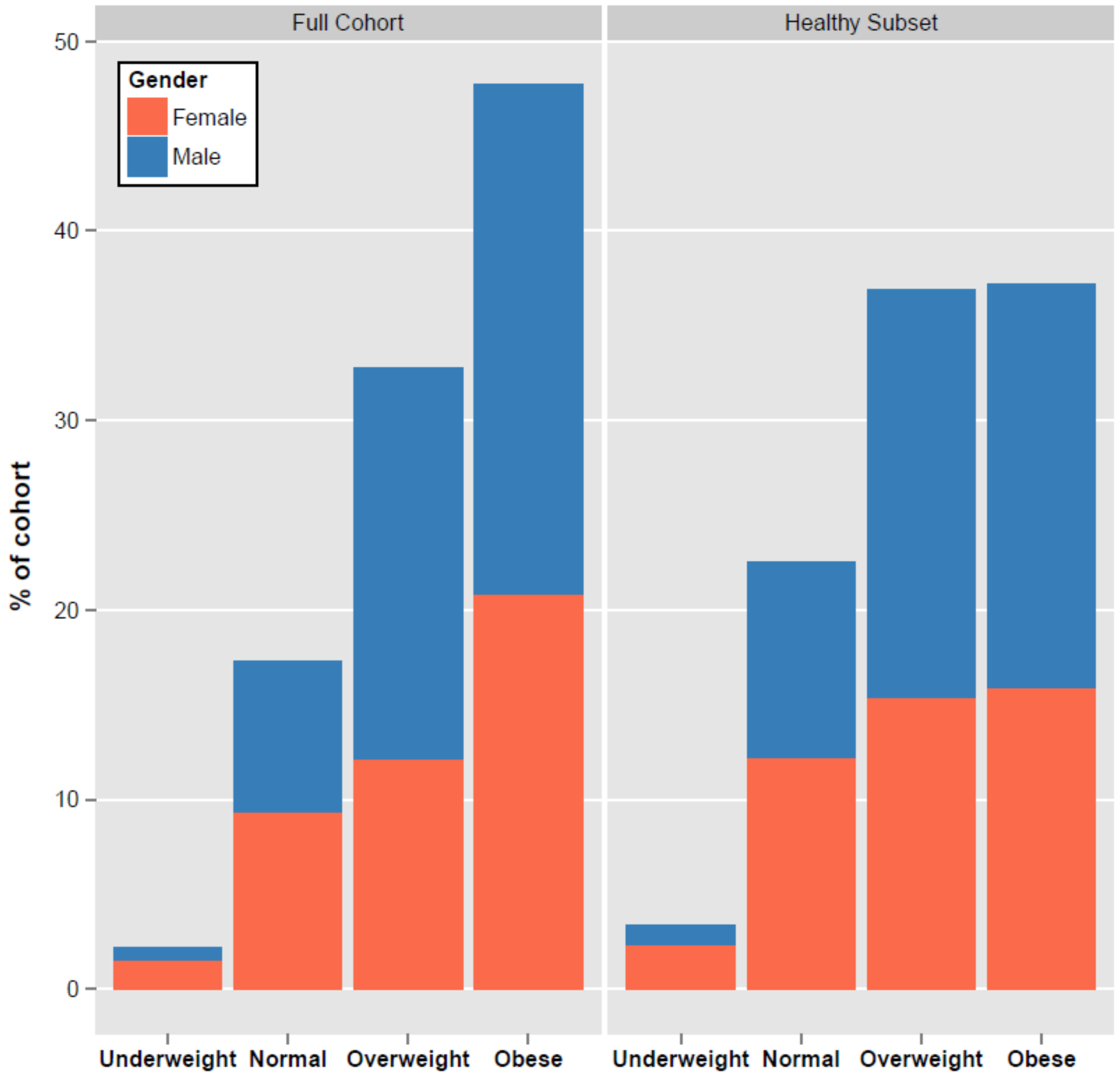

Figure S1: BMI stratification. Entire cohort (left), and a reduced cohort (right) comprising individuals without diseases* and with systolic BP of 100-139 and diastolic BP of 50-89 mmHg.

*based on questionnaire data

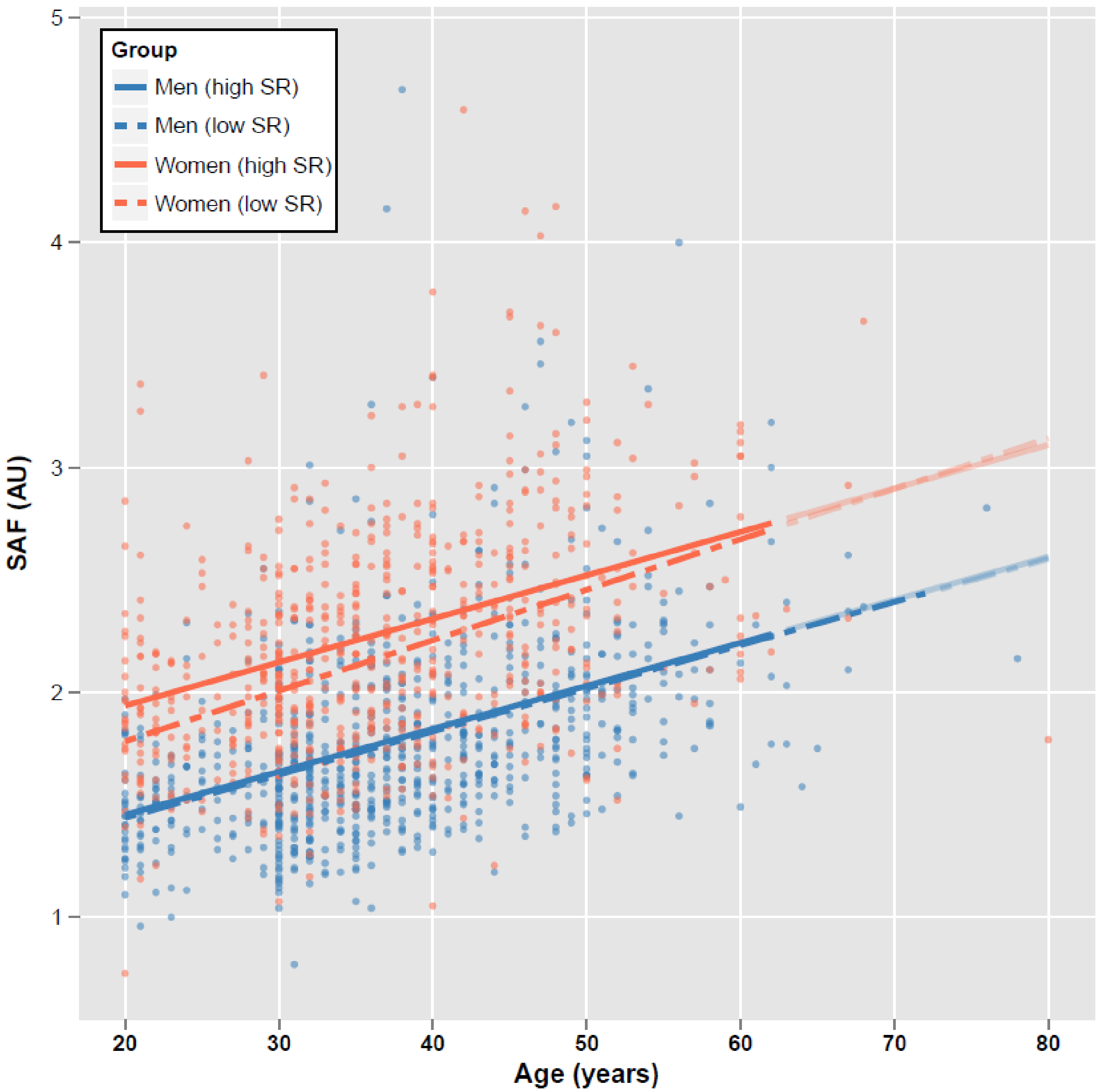

Figure S2: SAF vs age of a healthy sub-cohort, stratified by gender and SR. SAF increases with age, whereas women had higher SAF values over the entire age range than men. Study participants are categorized into two groups i.e. high SR (>10%) and low SR (<=10%). Women with higher SR showed higher SAF over time. Linear models were established with individuals up to an age of 60, and then extrapolated (transparent lines).

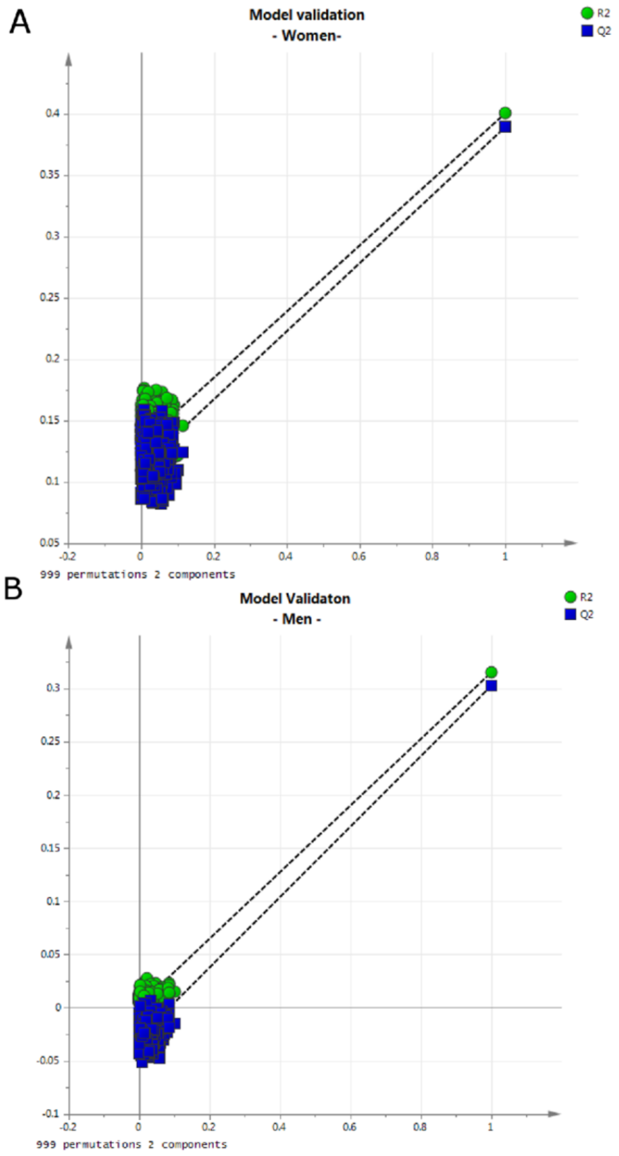

Figure S3: Orthogonal partial least squares regression (OPLSR) model validation for (A) women and (B) men. Plotted are model performance measures (R2 and Q2) from 999 permutation models and the non-permuted model over the similarity of the non-shuffled/non-permuted and shuffled/permuted SAF values.

Supplementary Tables:

Table S1: Change of SAF and SR after the application of skin lotion and subsequent skin washing.

Subject (Gender/ Age)	SAF* (AU)		SR* (%)	
	Δ cream[1] [%]	Δ wash[2] [%]	Δ cream[1] [%]	Δ wash[2] [%]
1 (M/40)	+26.3	-7.8	-23.7	+5.1
2 (F/33)	-2.2	+2.0	+13.1	-2.5
3 (M/39)	+18.6	+23.5	-13.9	-10.0
4 (M/29)	+17.9	+8.1	-19.5	-20.7
5 (M/35)	+20.1	+9.7	-17.8	-7.9
6 (M/34)	+23.4	+7.4	-27.6	-7.2
7 (F/32)	+5.6	+13.7	-1.5	+3.0
8 (F/33)	+36.4	-1.5	-11.8	16.1
9 (F/29)	+20.1	9.1	+3.7	-4.6
Average (±SD)	+18.5 (±10.6)	+7.1 (±8.4)	-11.0 (±12.7)	-3.8 (9.6)

* Based on averages of three successive measurements.

[1] Change of SAF/SR five minutes after the application of skin lotion (referenced to baseline values),

[2] Change of SAF/SR after application of skin lotion followed by skin washing with soap and water (referenced to baseline values).

Table S2: Intra-operator variation of SAF and SR.

Subject (Gender/Age)	SAF* (AU)			SR* (%)		
	Average (±SD)	CV [%]	Average CV (±SD)	Average (±SD)	CV [%]	Average CV (±SD)
**1** (M/40)	1.2 (±0.10)	8.5	6.2 (±2.0)	9.8 (±0.82)	8.4	8.1 (± 1.6)
**2** (F/33)	1.8 (±0.08)	4.5		29.4 (±2.17)	7.4	
**3** (M/39)	1.6 (±0.15)	9.5		11.1 (±0.98)	8.8	
**4** (M/29)	1.1 (±0.05)	4.4		8.0 (±0.39)	4.9	
**5** (M/35)	1.6 (±0.09)	5.5		10.1 (±0.89)	8.8	
**6** (M/34)	1.4 (±0.09)	6.6		8.9 (±0.88)	9.9	
**7** (F/32)	1.7 (±0.07)	4.2		9.0 (±0.74)	8.2	

* SAF/SR values represent averages of nine AGE reader measurements on the same subject by a single operator. Measurements were performed by the same operator on all seven subjects.

Table S3: Operator variation of SAF measurements.

Subject (Gender/ Age)	SAF (AU)					
	Operator 1*	Operator 2*	Operator 3*	Average (±SD)	CV [%]	Average CV (±SD)
**1 (M/33)**	1.17	1.19	1.21	1.19 (±0.015)	1.3	3.9 (±3.8)
**2 (M/35)**	1.41	1.50	1.48	1.46 (±0.040)	2.7	
**3 (F/29)**	1.62	1.70	1.56	1.63 (±0.058)	3.6	
**4 (F/51)**	2.29	2.35	2.27	2.30 (±0.034)	1.5	
**5 (F/53)**	3.01	2.98	2.85	2.95 (±0.067)	2.3	
**6 (M/33)**	1.59	1.33	1.80	1.57 (±0.193)	12.3	

* SAF values represent averages of three successive measurements

Table S4: Operator variation of SR measurements.

Subject (Gender/ Age)	SR (%)					
	Operator 1*	Operator 2*	Operator 3*	Average (±SD)	CV [%]	Average CV (±SD)
1 (M/33)	11.1	10.1	10.5	10.6 (±0.50)	4.8	4.6 (±2.3)
2 (M/35)	12.0	10.5	10.8	11.1 (±0.79)	7.1	
3 (F/29)	13.6	13.5	13.8	13.6 (±0.15)	1.1	
4 (F/51)	24.9	24.2	25.3	24.8 (±0.56)	2.3	
5 (F/53)	14.7	16.1	15.4	15.4 (±0.70)	4.5	
6 (M/33)	7.6	8.8	7.8	8.1 (±0.64)	7.9	

* SR values represent averages of three successive measurements
